# Neurological consequences of SARS-CoV-2 infections in the pediatric population

**DOI:** 10.3389/fped.2023.1123348

**Published:** 2023-02-14

**Authors:** Manon Casabianca, Caroline Caula, Luigi Titomanlio, Léa Lenglart

**Affiliations:** ^1^ Pediatric Emergency Department, APHP - Hopital Robert Debré, Paris Cité University, Paris, France; ^2^ Pediatric Migraine and Neurovascular Diseases Unit, APHP - Hopital Robert Debré, Paris Cité University, Paris, France; ^3^ DHU Protect, INSERM U1141, Paris Cité University, Paris, France

**Keywords:** SARS-CoV-2, encephalitis, children, COVID-19, anosmia

## Abstract

COVID-19 in the pediatric population is mostly asymptomatic. However, 1 out of 5 children presents non-specific neurologic symptoms such as headache, weakness, or myalgia. Furthermore, rarer forms of neurological diseases are increasingly being described in association to a SARS-CoV-2 infection. Encephalitis, stroke, cranial nerves impairment, Guillain-Barré syndrome or acute transverse myelitis have been reported and account for around 1% of pediatric COVID-19 cases. Some of these pathologies may occur during or after the SARS-CoV-2 infection. The pathophysiological mechanisms range from direct invasion of the central nervous system (CNS) by SARS-CoV-2 itself to postinfectious immune-mediated CNS inflammation. In most cases, patients presenting neurological pathologies related to SARS-CoV-2 infection are at greater risk of life-threatening complications and should be closely monitored. Further studies are needed to acknowledge the potential long-term neurodevelopmental consequences of the infection.

## Introduction

According to the world health organization (WHO), pediatric cases of COVID-19 account for up to 8.5% of cases in 2020. However, this proportion turns out to be much higher in some countries such as the United States where children represent 18.5% of cases (1, 2). Given the absence or poor symptoms of COVID-19 in this population, this prevalence is probably still underestimated (3–5).

When symptomatic, the most common symptoms are fever, cough and rhinorrhea (6) and some gastrointestinal symptoms such as diarrhea, vomiting or abdominal pain, can be present as well (6). The main reported complication related to SARS-CoV-2 infection in children is the multisystem inflammatory syndrome (MIS-C). The most recent definition of MIS-C is an illness in a person aged <21 years associated to (i) a subjective or documented fever, (ii) a clinical severity requiring hospitalization or resulting in death, (iii) the evidence of a systemic inflammation and (iv) new onset manifestations in at least two of the following categories (Shock, cardiac, mucocutaneous, gastrointestinal or hematologic involvement) in the absence of a more likely alternative diagnosis (7). These clinical criteria must be associated to laboratory confirmation of a SARS-CoV-2 infection to confirm the diagnosis of MIS-C (Table 1).

**Table 1:** Council of state and territorial epidemiologists/CDC surveillance case definition for multisystem inflammatory syndrome (MIS-C) in children associated with SARS-CoV-2 infection – June 2022 (7).

**Case definition classifications** **Confirmed:** Meets the clinical criteria and the laboratory criteria. **Probable:** Meets the clinical criteria and the epidemiologic linkage criteria. **Suspect:** Meets the vital records criteria.
**Clinical criteria** An illness in a person aged <21 years characterized by all of the following, in the absence of a more likely alternative diagnosis: • Subjective or documented fever (temperature ≥38 °C) • Clinical severity requiring hospitalization or resulting in death • Evidence of systemic inflammation [indicated by C-reactive protein of ≥3.0 mg/dl (30 mg/L)] • New onset manifestations in at least two of the following categories: • Cardiac involvement (indicated by left ventricular ejection fraction of <55%; coronary artery dilatation, aneurysm, or ectasia; or troponin elevated above laboratory normal range, or indicated as elevated in a clinical note) • Mucocutaneous involvement (indicated by rash, inflammation of the oral mucosa [e.g., mucosal erythema or swelling, drying or fissuring of the lips, strawberry tongue], conjunctivitis or conjunctival injection [redness of the eyes], or extremity findings such as erythema [redness] or edema [swelling] of the hands or feet) • Shock • Gastrointestinal involvement (indicated by abdominal pain, vomiting, or diarrhea) • Hematologic involvement (indicated by platelet count of <150,000 cells/*μ*l or absolute lymphocyte count of <1,000 cells/μl)
**Laboratory criteria** • Detection of SARS-CoV-2 RNA in a clinical specimen^§^ up to 60 days before or during hospitalization, or in a postmortem specimen using a diagnostic molecular amplification test (e.g., polymerase chain reaction); or • Detection of SARS-CoV-2–specific antigen in a clinical specimen^§^ up to 60 days before or during hospitalization, or in a postmortem specimen; or • Detection of SARS-CoV-2–specific antibodies^¶^ in serum, plasma, or whole blood associated with current illness resulting in or during hospitalization
**Epidemiologic linkage criteria** Close contact with a confirmed or probable case of COVID-19 disease in the 60 days before hospitalization.
**Vital records criteria** A death of a person aged <21 years whose death certificate lists MIS-C or multisystem inflammatory syndrome as an underlying cause of death or a significant condition contributing to death.

Neurological complications have also been described, in both adults and children (8–10). They can be differentiated into (i) non-specific neurological symptoms, like headache, weakness, myalgia or (ii) specific central or peripheral nervous system pathologies, like encephalitis, cranial nerves impairment, stroke, or Guillain-Barré syndrome (10–13). Non-specific neurological symptoms have been reported in up to 22% of pediatric patients. Specific neurologic pathologies account for 1% of cases (10, 12, 14–16) and are generally associated to more severe forms of COVID-19 (14, 17).

The aim of this manuscript is to present the different pathophysiological pathways underlying the neurological consequences of SARS-CoV-2, describe their clinical presentations and discuss therapeutics and prevention.

## Pathophysiological hypotheses

The mechanisms underlying the pathophysiological pathways of neurological symptoms of SARS-CoV-2 infection are still under study. However, a number of hypotheses have already been put forward. Some may explain (i) the occurrence of neurologic pathologies during acute SARS-CoV-2 infection, while others (ii) the neurologic pathologies showing after recovery.

### Neurological consequences occurring during the SARS-CoV-2 infection

The neurological symptoms occurring during the SARS-CoV-2 infection seem to be related to two different mechanisms: the direct invasion of the central nervous system (CNS) by the virus and an abnormal host inflammatory response.

The direct invasion of the CNS is possible by hematogenous or neuronal dissemination. In both cases, the spike protein of the virus binds both angiotensin-converting enzyme 2 (ACE2) receptors or transmembrane serine protease 2 (TMPRSS2) to infect the cells (18–20). In the hematogenous pathway, the virus first infects the respiratory tract, and then, from the bloodstream it crosses the blood-brain barrier (BBB) to enter the CNS. Once the BBB is disrupted, leukocytes infected by the SARS-CoV-2 can pass through, leading to their dissemination in the CNS and subsequent inflammation. Regarding the viral dissemination by retrograde axonal transport, the main hypothesis is that SARS-CoV-2 infects the olfactory receptor neurons (the only part of the CNS that is not protected by the dura), crosses the neuroepithelium reaching the olfactory bulb and then invades the brainstem, cortex and basal ganglia *via* the nerves (8, 10, 13, 19–21). The direct invasion of the CNS, leading to encephalitis, is however probably infrequent. Indeed, PCR of SARS-CoV2 performed in CSF when patients presented neurologic complications yielded positive in a very small proportion of cases (6% in a systematic review reporting 304 CSF studies) (22, 23). This may be due to the low sensitivity of PCR as it is currently performed, or to the fact that the SARS-CoV-2 has a weak direct neurotrophic effect.

Strokes and vasculitis have also been described during an acute SARS-CoV-2 infection (10, 19, 24, 25). They could be the consequence of an aberrant host inflammatory response to the virus. Indeed, it has been observed that coronaviruses, particularly SARS-CoV-2, trigger molecular mechanisms that interfere with the host adaptative immune response (ineffective IFN response, reduction of lymphocyte count etc) (26). Moreover, the association with a pro-coagulable state and endothelial dysfunction (vascular disruption, activation of the clotting cascade by exposure of thrombogenic activator) increases the risk of thrombotic complications.

### Neurological consequences occurring after the SARS-CoV-2 infection

In this case, too, two hypotheses are to be considered. The first one is based on a retarded multisystemic inflammatory response to the viremia. This uncontrolled inflammatory state leads to the multi-organ damages that have been described in MIS-C. The release of inflammatory agents leads to a cytokine storm causing the disruption of the integrity of the BBB allowing various molecules (tumor necrosis factor alpha (TNF-α), interleukin (IL)-1β, IL-6, IL-12, and interferon-gamma (INF*γ*)), as well as infected and immunes cells to penetrate into the CNS (10–13, 25, 27). Neurologic complications during MIS-C seems to be both non-specific and specific neurologic pathologies (13, 28–30).

The second hypothesis relies on postinfectious immune-mediated mechanisms: SARS-CoV-2 might induce an autoimmune response by activating antibodies against brain components or *via* “molecular mimicry”: the spike protein of the SARS-CoV-2 could cross-reactive with CNS components explaining some of the neurologic complications (10, 12, 19, 27, 31–33). Indeed, the SARS-CoV-2 spike protein binds not only to the ACE-2 receptor but also to sialic acid-containing glycoproteins and gangliosides on cell surfaces. There could be a cross-reactivity between components of the spike protein binding normally to gangliosides and some of surface peripheral nerve glycolipids residues. The molecular mimicry could explain reported post-infectious pathologies like Guillain Barré-syndrome (GBS) or acute disseminated encephalomyelitis (ADEM) (19, 34).

Besides, as in many viral infections, neurological manifestations can be caused or aggravated by hypoxemia, septic shock, metabolic or electrolyte disorders that COVID-19 infection can induce (10, 12, 27, 35).

### Particularities of children's immune responses to SARS-CoV-2

It has been well documented and described that children present less severe forms of SARS-CoV-2 than adults. The mortality rate for children has been estimated under 0.1% with about 2% of children admitted to intensive care units (36). Different factors may contribute to this difference. First of all, children usually have no comorbidities, so, they do not suffer from the baseline pro-inflammatory state (increased by hypertension, smoking, hypercholesterolemia, obesity etc.) more commonly observed in aging adults (26). Secondly, the host immune response in children is more efficient than in adults (26) (increased number of adaptive immunity cells CD4+/CD8+, better functional capacity of B-cells) and the trained innate immunity (37) (frequent exposure to antigens as vaccine or infections) increases its baseline reactivity to pathogens. These particularities may explain the smaller number of severe COVID-19 cases in children, but they do not completely prevent immune dysregulation mediated by the coronaviruses and its consequence.

## Clinical consequences of SARS-CoV-2 infection

### Encephalitis, meningoencephalitis

Encephalitis is an inflammation of the brain causing neurologic dysfunction. Etiologies are multiples, as reported above: direct invasion, post-infectious pathologies such as acute disseminated encephalomyelitis (ADEM), or non-infectious condition such as N-methyl-D-aspartate receptor (NMDAR) encephalitis. Non-autoimmune encephalitis could concern between 7.5% and 49% of hospitalized adult COVID-19 patients. No incidence has been established in children due to its low incidence (38), however, many case reports have been described (Table 2). The main symptoms of meningoencephalitis due to COVID-19 infection are seizures (29.5%), confusion (23.2%), headache (20.5%), alteration of mental status (11.6%) (39). Psychotic symptoms, cerebellar ataxia and focal deficit have also been described but they seem rarer (8, 19, 39). In most cases, when the study of the CSF has been carried out, the RT-PCR in search of SARS-CoV-2 was negative, or a non-specific lymphocytic pleocytosis could be found (11, 40). Cases of N-methyl-Aspartate receptor (NMDAR) encephalitis as well as MOG antibody-associated encephalitis have been described (39, 41–45).

**Table 2:** Summary of frequencies of neurological pathologies related to SARS-CoV-2 in children and in adults, and most probable pathophysiological mechanisms.

Neurological pathologies related to SARS-CoV-2	Reports in children	Frequency in adults	Most probable patho-physiological mechanisms
Encephalitis, meningoencephalitis	Case reports (50 < *n* < 100)	Encephalitis: 7.5–49% of hospitalized patients Auto-immune encephalitis (ADEM, NMDA-R): Case reports 50 < *n* < 100)	- Brain parenchymal invasion by olfactory nerve, - Hematogenous spread, - Systemic inflammation with blood brain barrier disruption, - Post-infectious immune-mediated
Stroke	0.32, up to 1%	1%, up to 3%	- Systemic inflammation with endothelial dysfunction, - Coagulopathy
Cranial nerve impairment	Anosmia, ageusia: 0.5%, up to 2% Other cranial nerve impairment: case report	Anosmia, ageusia: up to 86% Other cranial nerve impairment: case report	Direct invasion of the olfactory, third and vestibular nerves
Guillain-Barré Syndrome	Case reports (10 < *n* < 50)	0.015%	Post-infectious immune-mediated
Acute Transverse Myelitis	Case reports (*n* < 10)	Case reports (50 < *n* < 100)	- Systemic inflammation with blood brain barrier disruption, - Post-infectious immune-mediated

Brain MRI studies are reported for only a few children, and are usually normal. Some authors report T2-hyperintense lesions associated with restricted diffusion in the splenium of the corpus callosum (SCC) (14, 15, 19, 46). These lesions seem to be more frequent when encephalitic symptoms occurs during MIS-C, and they are similar to the ones described in other inflammatory syndromes such as Kawasaki disease with reversible encephalopathy (47, 48). Comparing to the adult population with encephalitis, Ellul and al report 6 out of 8 cases with a normal MRI, while two other studies report between 44% and 100% of abnormal MRI, with FLAIR signals in the frontotemporal cortex (8, 31, 49). Electroencephalogram (EEG), when performed, shows nonspecific abnormalities such as diffuse slowing. Focal abnormalities are rarely reported, particularly in cases of encephalitis associated with seizures. In the great majority of cases, the EEG normalized within 2 weeks (8, 11–13, 31, 50).

The prognosis of encephalitis during a SARS-CoV-2 infection is quite hard to define. Only a few cases have been described, and they are quite heterogeneous: with or without a positive PCR in the CSF, presence or absence of NMDAR antibodies, normal or abnormal neuroimaging, treated or not by steroids and so on. Some studies report a complete recovery (19, 41, 50, 51) whereas others report an unfavorable outcome (14).

Cases of ADEM post-COVID do not clinically differ from ADEM post other viruses (41, 48, 52–55). So, the encephalitic presentation described above is enriched by focal symptoms: motor deficit, loss of reflexes or hyperreflexia, impairment of cranial nerves. Moreover, acute hemorrhagic necrotizing encephalitis have also been reported (39, 56, 57). All patients (adults and children) had a fatal outcome, except for a recent report of an 11-year-old boy treated with tocilizumab (57).

An ICU monitoring seems mandatory in COVID-19 encephalitis because of a great risk of life-threatening complications (12–14).

### Stroke

SARS-CoV-2 infection - as other viruses - with its degree of coagulopathy, inflammation and endothelial damage can cause secondary cerebral arteriopathy and lead to stroke (41). According to studies, 0.32 to 1% of children admitted for COVID-19 presented stroke, compared to 1 to 3% of adults (8, 58–62) (Table 2).

The majority of the described pediatric cases presented risk factors for strokes (14, 35, 41, 58) such as cardiac disease, arteriopathy/arteritis, or prothrombotic disorders. However, LaRovere et al. (14) described 4 healthy children out of 12 stroke cases, for which, COVID-19 seemed to be the only causative factor. A recent review of 23 pediatric cases found similar results (62). Interestingly, even asymptomatic children could suffer from strokes, suggesting that the aberrant host immune response would contribute to increase the risk of coagulopathy, thrombocytopathy and endotheliopathy conducting to strokes (63) even in non-symptomatic cases (64, 65). Indeed, more than 80% had a biological inflammatory syndrome and all presented either arteritis or focal arteriopathy on brain MRI. Elevated D-Dimer appeared as an independent biomarker for SARS-CoV-2-related ischemic stroke in adults and as a risk factor of thrombotic-events in children (62, 64). D-Dimer, could be a parameter to monitor whilst SARS-CoV-2 infection as a stroke predictive factor.

Regarding the prognosis for these patients, it mainly depended on the size and localization of the stroke, as well as the presence of risk factors.

### Cranial nerve impairment

The loss of smell and taste is commonly associated with SARS-CoV-2 infection in the adult population (8, 31). The pathophysiology is not yet fully understood but is likely associated to direct olfactory invasion (12, 66). Its prevalence has been estimated at its highest to 86%, but varies widely according to studies. This could be partly explained by the subjective nature of this symptom (8). In children compared to adult, this complication seems to be less frequent, but it is very difficult to diagnose, if not impossible under a certain age (13, 21, 61–64). Thus, the incidence in children has been estimated to be around 0.5% to 2% and is probably under-reported (10, 14, 16) (Table 2).

Explorations carried out when children presented a loss of taste or smell are practically non-existent, and the few cases reporting brain MRI results do not seem to show any anomaly of the olfactory region (48, 67). Some studies in adults have shown an increased olfactory bulb size with a pathological signal at MRI (68–70).

The prognosis is usually good, and symptoms disappear within 1 to 2 weeks. In some cases, they can persist after other symptoms have resolved. Treatments for dysgeusia are rarely reported. Several treatments have been tested for anosmia: saline irrigations, nasal or oral corticosteroids (67, 71). The only one that seems to have shown its effectiveness in adults, is olfactory rehabilitation (72).

Vestibular neuritis as well as facial nerve palsy have been reported in children during a COVID-19 infection (73–75). SARS-CoV-2 could explain the increase of facial nerve palsy cases during the pandemic in both adults and children (76–78). Other cranial nerve palsies, with enhancement of the nerve at brain MRI, have been described (48). Particularly, cases of third cranial nerve palsy (79–81) revealed by unilateral diplopia and ptosis have been described with good outcomes after corticosteroid treatment.

### Guillain-Barré syndrome (GBS)

Guillain-Barré syndrome is a post-infectious immune-mediated polyneuropathy. The estimated incidence of GBS is 15 cases per 100,000 SARS-CoV-2 infections (82). As previously described for GBS not related to COVID-19 infection, male gender appears to be a risk factor even in the pediatric population (82–84) (Table 2). The exact pathophysiology has not been elucidated yet (19, 35). The syndrome may appear between a few days and several weeks after a SARS-CoV-2 infection (11). According to the reported cases, the clinical presentation is similar to non-COVID 19 related cases: rapid ascending bilateral decrease of the motor force, diminished or abolished tendon reflex and distal paresthesia. Dysautonomia and pain may accompany the course of the disease. Electrophysiological studies have shown acute inflammatory demyelinating polyneuropathy. MRI of the spine showed abnormal enhancement of the nerve roots. CSF analysis confirmed the cytological dissociation of albumin (42, 55, 85–89).

The outcome after treatment with intravenous immunoglobulin varies between a total recovery and the persistence of a severe handicap or even death. A systematic review carried out in an adult population reported more than 70% of patients with a good prognosis (90). It seems that COVID-19 does not influence the prognosis of the disease (82).

### Acute transvers myelitis (ATM)

The pediatric literature reports rare but typical cases of transverse myelitis in patients with no medical history (Table 2) and in whom SARS-CoV-2 infection seemed to be the only triggering event (91–94). This complication occurs during or longer after the SARS-CoV-2 infection and has been more often described in adults (95). The clinical presentation is characterized by the evolution towards flaccid quadriparesis with areflexia. Involvement may be cervical, thoracic or extended longitudinally. MRI commonly finds a hyperintense T2 signal localized in the affected cord (48). Depending on the severity of the disease, the patients received boluses of methylprednisolone either alone or with plasma exchanges or immunoglobulin. Treatment aimed to prevent permanent disabilities in these case reports (94).

## Therapeutics and prevention

SARS-CoV-2 infection in children can be responsible for rare and various neurological consequences. In view of the different pathophysiologic mechanisms and the adult literature, there is little evidence supporting the interest of antiviral therapy in central or peripheral nervous system pathologies related to SARS-CoV-2 previously described (38). Indeed, neurological manifestations are mainly immune-mediated, so they should be better managed by anti-inflammatory (corticosteroids) or immunomodulatory treatments such as immunoglobulin, colchicine or tocilizumab (anti-IL6 antibodies) (96).

As for today, there is no reports, in the literature (97), of neurological consequences of the various SARS-CoV-2 vaccines in the pediatric population. Regarding immunogenicity, studies (98, 99) report very small numbers of MIS-C after receiving one dose or more of COVID-19 vaccine. Moreover, in the adult population, the incidence of GBS (100) or central veinous thrombosis (101) is lower after COVID-19 vaccination, supporting the safety and interest of the vaccine to prevent neurological consequences as well as severe forms of COVID-19 and the transmission of SARS-CoV-2.

## Conclusion

Since the beginning of the SARS-CoV-2 pandemic, the progressive increase of pediatric cases has raised attention on rare forms of COVID-19 that were not highlighted previously. Reports of neurological consequences of SARS-CoV-2 range from non-specific neurological symptoms to specific central or peripheral nervous system diseases like encephalitis, cranial nerves impairment, stroke or Guillain-Barré syndrome. The host immune response of children being more efficient and not interfered by a baseline pro-inflammatory state, these neurological consequences of SARS-CoV-2 seem to be less frequent in children than in adultes. In most cases, patients presenting neurological pathologies related to SARS-CoV-2 infection have poorer outcomes, and are at risk of life-threatening complications.

The long-term neurodevelopmental consequences after a neurological complication of COVID-19 are unknown and need further investigations.
